# Growth of Oxide and Nitride Layers on Titanium Foil and Their Electrochemical Properties

**DOI:** 10.3390/ma18020380

**Published:** 2025-01-15

**Authors:** Song Hyeon Kim, Young-Il Kim

**Affiliations:** Department of Chemistry, Yeungnam University, 280 Daehak-ro, Gyeongsan 38541, Republic of Korea; wise5649@naver.com

**Keywords:** supercapacitor, coating, ammonolysis, heterostructure, nitride

## Abstract

The surface of titanium foil can be modified by heating in the air, in a N_2_ flow, and in an NH_3_ flow. Upon heating in the air, the elemental Ti gradually transforms to Ti_3_O at 550 °C and to rutile TiO_2_ at above 700 °C. Treatment in a N_2_ flow leads similarly to Ti_3_O at 600 °C and TiO_2_ at 700 °C, although the overall reaction is slower. Meanwhile, nitridation in the N_2_ flow is minimal, even at 900 °C. Heat treatment in an NH_3_ flow produces nitride phases through the ammonolysis of the hexagonal Ti. With an ammonolysis at 900 °C, trigonal Ti_2_N and cubic TiN form together while, at higher temperatures, TiN is dominant. The TiN layer can also be obtained via the ammonolysis of the TiO_2_ coating, that is, by the sequential treatments of Ti in the air and then in an NH_3_ flow. The titanium nitride layers have particulate microstructures and varying degrees of porosity, depending on the ammonolysis temperature and time. The TiO_2_-derived TiN has a significantly higher capacitance than TiN derived directly from Ti. The optimally prepared TiN specimen exhibits an areal specific capacitance of 66.2 F/cm^2^ at 0.034 mA/cm^2^.

## 1. Introduction

Titanium can form binary oxides, nitrides, and oxynitrides with widely flexible compositions. Titanium dioxide with Ti^4+^ occurs mainly in three polymorphs, rutile, anatase, and brookite [1], among which rutile is the most stable phase in the ambient condition [2]. The above three polymorphs commonly consist of corner- and edge-shared TiO_6_ octahedra but are distinguished by an octahedral linkage pattern [1]. In addition, titanium has a rich chemistry of suboxides too. Most notably, the Magneli phases Ti*_n_*O_2*n*−1_ (*n* = 4–10) represent homologous members of reduced titanates and have been established since as early as 1957 [3]. The Magneli phases are also based on the TiO_6_ octahedra but have shear planes created by the face-sharing of octahedra. The reduction of cation greatly impacts the optical and electrical properties of Magneli phases, so they are often referred to as black titania [4,5,6]. Other reduced phases of titanium oxide include the compositions of Ti_3_O_5_, Ti_2_O_3_, TiO, and Ti_2_O [7,8]. Furthermore, titanium-rich phases Ti_3_O and Ti_6_O are formed by the interstitial dissolution of oxygen in the hexagonal lattice of titanium [9,10,11]. In the meantime, titanium nitrides and oxynitrides are reported in TiN, Ti_2_N, TiN_1−*x*_, Ti_3_N_2−*x*_, Ti_4_N_3−*x*_ [12,13,14,15,16], and TiO*_x_*N*_y_* [17,18]. While TiO_2_ has received enormous attention because of its photocatalytic and photovoltaic properties [19,20,21], the reduced titania have been of great interest owing to their potential for diverse application areas, such as in supercapacitors, fuel cells, photocatalysts, electrocatalysts, and sensors [4,5,6,7,8,22]. On the other hand, titanium nitrides and oxynitrides are attractive due to their excellent mechanical hardness [23,24], chemical stability [25], electronic conductivity [26,27,28], photocatalytic and electrocatalytic functions [29,30,31], and supercapacitor activity [31,32,33,34,35,36].

Thin film deposition is an important part of materials research and engineering, and it often constitutes an essential step in manufacturing the device products. Indeed, for all the aforementioned applications of titanium compounds, active materials have to be coated on a substrate of choice. For instance, the working electrodes of the electrochemical system are prepared by properly coating the current collector with an electroactive material. It is important, in such coatings, to achieve a high-quality adhesion between the active material and the substrate for realizing the desired performance [37,38]. Drop casting [27,36,39,40,41] is perhaps the simplest and most popular method for preparing electrochemical electrodes and can be applied to virtually any powder material, but it is generally perceived as providing an inferior coating to more sophisticated methods such as electrochemical synthesis [34,42], physical vapor deposition [38,43], chemical vapor deposition (CVD) [44,45], and atomic layer deposition (ALD) [46], which use a metallic object as the substrate surface.

In this regard, we investigated two things by examining the thermally induced conversion of Ti foil in atmospheres of air, N_2_, and NH_3_. First, we explored the formations of various nitrides and oxides of titanium, as functions of the gas atmosphere, temperature, and time of the heat treatment. We expected that, by using the dense metal foil as the parental reactant, kinetically favored metastable phases could be produced because of the restricted diffusion of the reacting gases. Second, through the thermal conversion of Ti foil, we attempted to develop superior electrode structures, where the functional compound is deposited on the conductor with a desirable interface contact. This approach is advantageous, as it can provide an autogenous coating of the binary Ti compounds on the metallic substrate through a simple process. Here, we present the stability conditions of different oxide and nitride phases of Ti and demonstrate that the Ti foil-derived specimens can be conveniently used as the electrode for electrochemical processes.

## 2. Experimental Procedure

Titanium foil was used as the starting material for obtaining titanium oxides and nitrides. As-purchased Ti foil (Alfa Aesar (Ward Hill, MA, USA), 99.99%, thickness 0.127 mm) was cut into 15 mm × 15 mm squares, immersed in 1 N HNO_3_ for 30 min, rinsed with deionized water, and dried. Then, the Ti foil was heated in static air, N_2_ flow (Shumachemia (Yeongcheon, Republic of Korea), 99.999%), or NH_3_ flow (PSG Co. (Pusan, Republic of Korea), 99.9999%), where the temperature and time were varied at 500~1000 °C and 2~60 h, respectively. The gas flow was maintained at a rate of ≈200 sccm in all cases. For comparison, syntheses of Ti oxides and nitrides were examined using Ti powder (Kojundo (Sakado, Japan), 99.9%) and TiO_2_ powder (Alfa Aesar (Ward Hill, MA, USA), 99.995%; rutile) too.

The powder X-ray diffraction (XRD) pattern was collected in the Bragg–Brentano mode using a laboratory diffractometer (Rigaku (Akishima, Japan), MiniFlex 600) with Cu *K*_α_ radiation (40 kV, 15 mA). The plane and fracture surfaces of the foil sample were examined using optical microscopy and scanning electron microscopy (SEM; Hitachi (Tokyo, Japan), S-4800). Ultraviolet–visible (UV–Vis) spectra were collected in a diffuse–reflectance spectrometer (Scinco (Seoul, Republic of Korea), Neosys 2000) equipped with a 35 mm integrating sphere, using BaSO_4_ as a reference.

The electrochemical properties were tested using cyclic voltammetry (CV) and galvanostatic charge–discharge (GCD) in a potentiostat/galvanostat (AMETEK (Oak Ridge, TN, USA), VersaSTAT 3), using a three-electrode cell employing 1 M KOH(*aq*) as an electrolyte. Titanium nitride-coated foil, Pt coil, and Ag/AgCl were used as the working, counter, and reference electrodes, respectively. The working electrode was constructed using a custom-made sample holder that exposed only one side of the foil specimen (active area 0.88 cm^2^). Both CV and GCD were carried out in the potential (*E*) range of −0.2 + 0.5 V vs. Ag/AgCl. The areal specific capacitance, *C*_s_ in F/cm^2^, was calculated as ∫I dE/(v×∆E×A) from CV, and (I×∆t)/(∆E×A) from GCD, where ∫I dE, v, ∆E, A, and ∆t are integral CV area, potential scan rate, potential window width, electrode area, and time length, respectively [38].

## 3. Results

### 3.1. Formations of Titanium Dioxide and Suboxide Coatings

**Figure 1:**
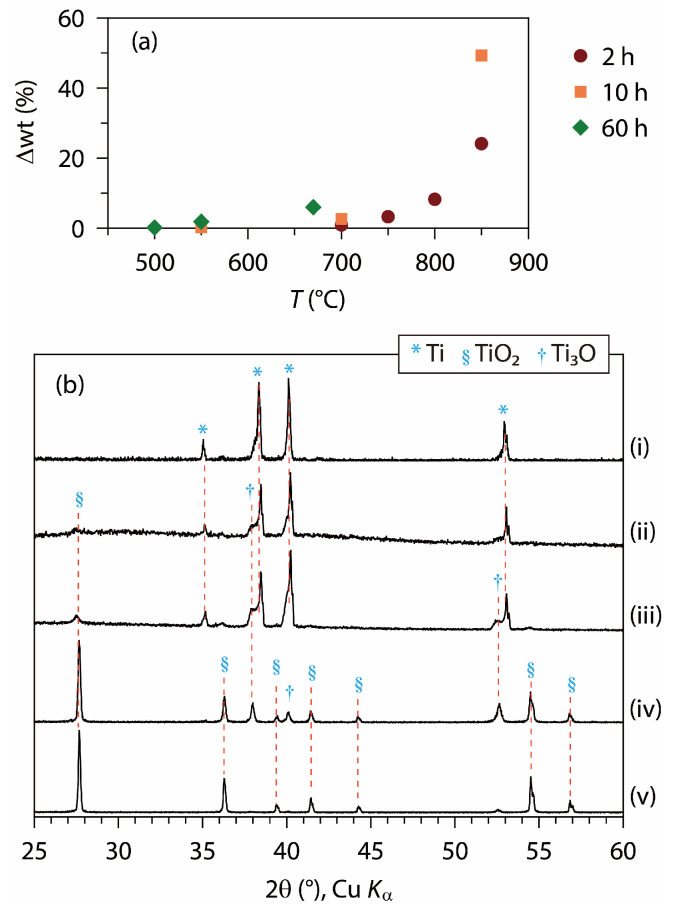
(**a**) Weight change (Δwt) and (**b**) XRD patterns of heat-treated Ti foils in air, depending on temperature and time: (i) pristine Ti foil, (ii) 500 °C, 60 h, (iii) 550 °C, 10 h, (iv) 700 °C, 2 h, (v) 800 °C, 2 h.

Elemental titanium is readily oxidized when heated in the air. Thus, the Ti foil was transformed into the titanium dioxide or suboxides upon heat treatment in the air. The extent of oxidation could be estimated using Ti foil’s weight change (Δwt) upon heat treatment. For example, a complete conversion of Ti (FW 47.87) to TiO_2_ (FW 79.87) corresponds to a Δwt of + 66.8%. Figure 1a shows Δwt values observed after heating Ti foils in the air under various temperature and time conditions, and Figure 1b shows the XRD patterns of several samples. Comparing the relative XRD intensities of foil and powder samples, it was judged that the Ti foil was polycrystalline with random crystal orientations (Figure S1). As shown in Figure 1a, the oxide conversion was minimal at 500 °C but was expedited by the increases in temperature and time. With temperatures of 600 °C or below, Ti_3_O [9,47] was observed together with rutile TiO_2_ whereas, with higher temperatures, the rutile phase was increasingly dominant. Heat treatment at 800 °C for 2 h produced an almost pure rutile phase (Figure 1b, v) that exhibited similar XRD peak intensity ratios to those of a powder reagent (Figure S1). The other polymorphs of TiO_2_, however, were not detected, regardless of the temperature and time of heating. In ambient conditions, Ti has a hexagonal structure (*P*6_3_/*mmc*, *a* = 2.95 Å, *c* = 4.69 Å) [48] and rutile TiO_2_ is tetragonal (*P*4_2_/*mnm*, *a* = 4.59 Å, *c* = 2.96 Å) [49].

**Figure 2:**
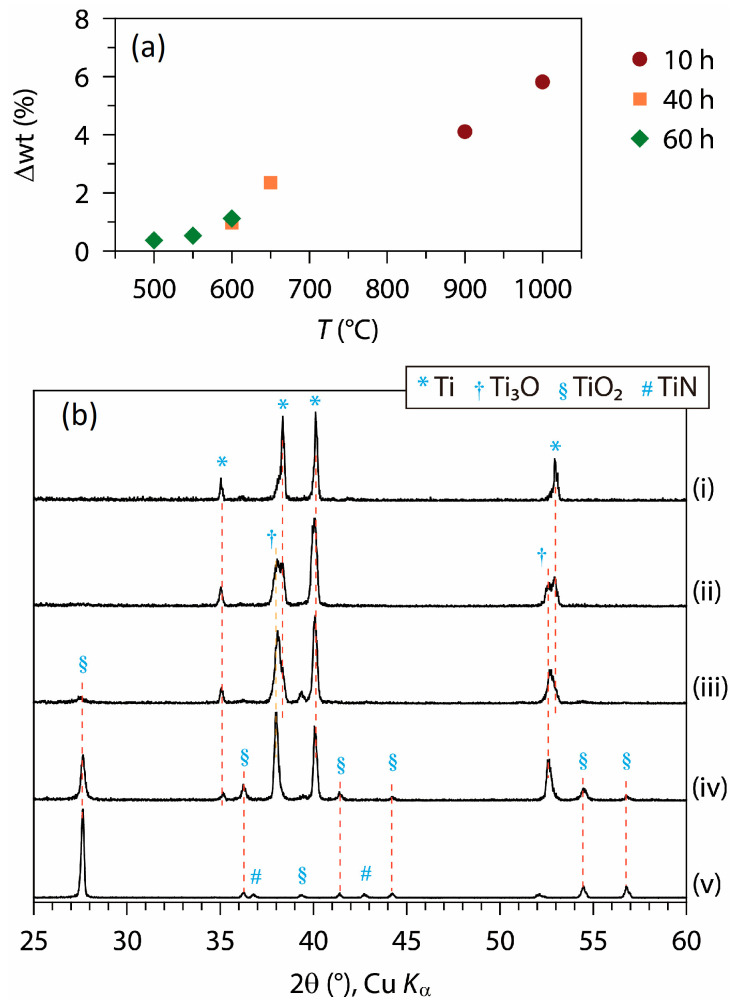
(**a**) Weight change (Δwt) and (**b**) XRD patterns of heat-treated Ti foil in N_2_, depending on temperature and time: (i) pristine Ti foil, (ii) 550 °C, 60 h, (iii) 600 °C, 60 h, (iv) 650 °C, 40 h, (v) 900 °C, 10 h.

The thermal reaction of Ti foil was investigated also in a N_2_ atmosphere. Figure 2a shows the Δwt values observed after heating Ti foils in a N_2_ flow using various temperature and time parameters, and Figure 2b shows the XRD patterns of select samples. Compared with heating in air, the Δwt values are much smaller for heating in N_2_. At temperatures above 650 °C, rutile TiO_2_ was formed as the main phase while, at 600~650 °C, Ti_3_O was dominant (Figure S2). When heated at 900 °C for 10 h, the rutile phase was obtained, along with a trace level of TiN (Figure 2b, v). The above TiO_2_ layer derived under N_2_ had a marked preferred orientation, as evidenced by the exaggerated intensity of the (110) peak at ≈27.6° 2θ.

The parental Ti has a hexagonal close-packed (*hcp*) structure, and its suboxides Ti_6_O and Ti_3_O are based on the *hcp* array of Ti wherein O partially fills the octahedral hole in an ordered manner [9]. Therefore, Ti_6_O and Ti_3_O are considered intermediate phases formed in the early stage of the oxide conversion of Ti. The introduction of interstitial oxygen expands the *c*-axis of Ti but has little influence on the *a* parameter. Accordingly, the gradual oxidation of Ti was accompanied by discernible shifts of the (002) peak at ≈38° 2θ and the (012) peak at ≈53° 2θ. On the other hand, the (010) peak position was nearly unchanged at ≈35° (Figure S2). Previously, the above titanium-rich suboxides Ti_6_O and Ti_3_O were prepared via arc melting of the TiO_2_/Ti mixture, followed by annealing for equilibration [9,10,11]. The present scheme, however, provides a much simpler route to Ti_3_O. N_2_ gas can be used for nitridation, and such reactions can be accomplished for highly reactive substances or when under extremely low O_2_ partial pressure. In the present study, N_2_ gas was used as purchased without further purification, and presumably contained a trace level of O_2_. The experimental results indicate that O_2_ is far more reactive than N_2_. Interestingly, the powder form of Ti, when heated in N_2_, could be nitrided to a pure TiN, demonstrating the far higher reactivity of the powdered form than the dense foil.

### 3.2. Formations of Titanium Nitride Coatings

**Figure 3:**
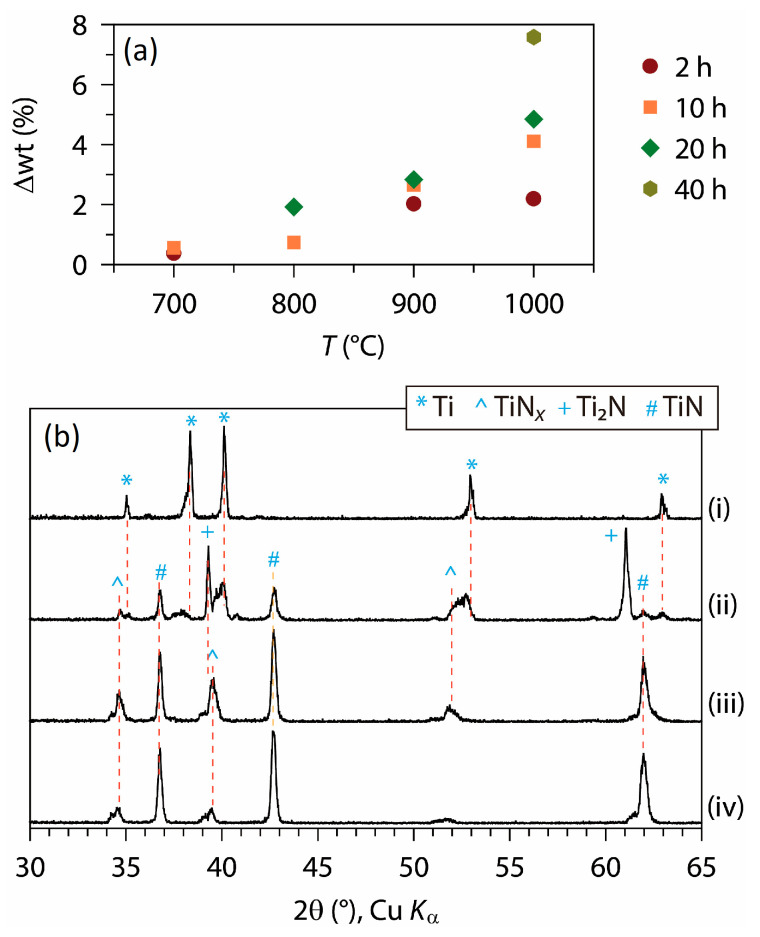
(**a**) Weight change (Δwt) and (**b**) XRD patterns of heat-treated Ti foil in NH_3_, depending on temperature and time: (i) pristine Ti foil, (ii) 900 °C, 2 h, (iii) 1000 °C, 2 h, (iv) 1000 °C, 10 h.

Ammonia is a much stronger nitriding agent than N_2_, and thermal ammonolysis is considered to be among the most efficient nitridation methods [50]. Figure 3a shows Δwt values observed after heating Ti foils in the NH_3_ flow, and Figure 3b shows the XRD patterns of nitridation products. The increases in temperature or time led to a higher level of nitridation, such that a nearly pure TiN phase (cubic, *a* = 4.24 Å) [12,51] was obtained upon heat treatment of Ti foil at 1000 °C for 10 h. Meanwhile, TiN*_x_* (*x* ≈ 0.3) [52] and Ti_2_N phases [53] were formed under milder conditions. The nitrided foils exhibited a metallic luster and various colors ranging from bright bronze to dark brown.

The titanium nitrides could also be obtained by heating a TiO_2_-covered foil in NH_3_. Figure S3 shows the XRD patterns corresponding to the sequential transformation of Ti foil to TiO_2_ to TiN. For comparison, the nitridations of Ti powder and TiO_2_ powder were examined in N_2_ and NH_3_. Unlike the foil form, Ti powder was fully nitrided to TiN in either the N_2_ or the NH_3_ flow (Figure S4), but the TiO_2_ powder was not reactive in the N_2_ atmosphere (Figure S3b).

### 3.3. Microscopic Images and Optical Properties

**Figure 4:**
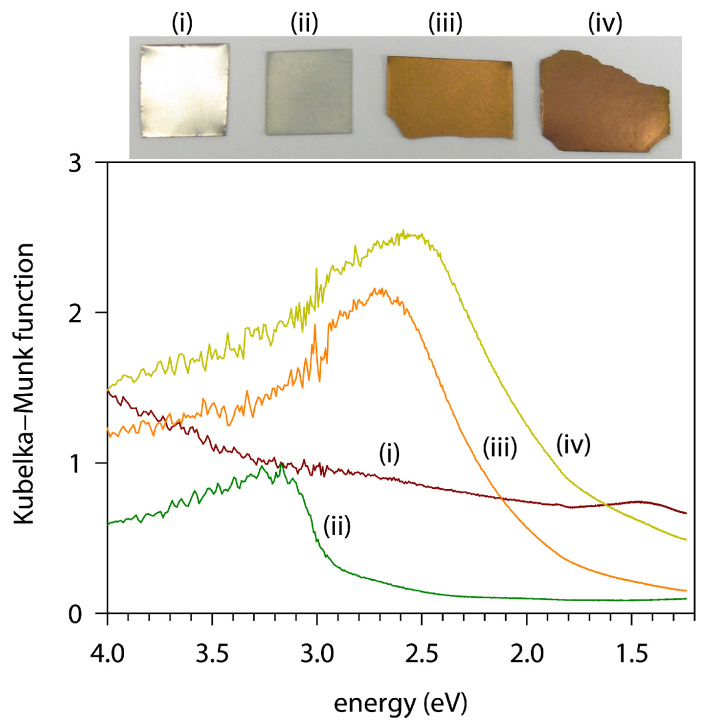
Photographs and UV–Vis diffuse–reflectance absorption spectra of (i) Ti foil and (ii–iv) coatings prepared using various heat treatments: (ii) 800 °C for 2 h in air, (iii) 1000 °C for 10 h in NH_3_, and (iv) 750 °C for 2 h in air and then 900 °C for 2 h in NH_3_.

Figure 4 shows the photographs of Ti foil and the nitride and oxide derivatives, together with the UV–Vis absorption spectra retrieved from the diffuse–reflectance following the Kubelka–Munk transformation [54]. The Ti foil showed a high reflectance over the entire visible light range and, consequently, a significant magnitude of Kubelka–Munk function. The TiO_2_-coated specimen (Figure 4, ii) was ivory, and exhibited a band gap energy of 2.9 eV, which was ≈0.1 eV smaller than that of bulk TiO_2_ powder (Figure S5). The nitridation products also showed a semiconductor-like absorption edge. The band gap energies were estimated at 1.9 eV for the TiN obtained directly from the Ti foil, and at 1.6 eV for the one obtained from the nitridation of the TiO_2_ layer.

**Figure 5:**
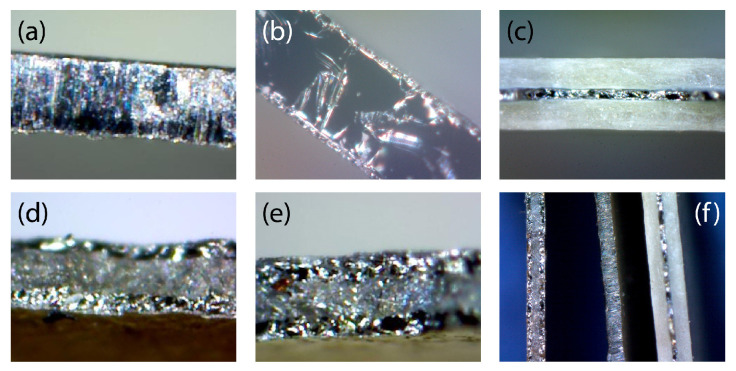
Optical microscopic images for the cross-sections of (**a**) Ti foil and (**b**–**e**) coatings prepared using various heat treatments: (**b**) titanium suboxide, (**c**) titanium dioxide, (**d**,**e**) titanium nitrides, and (**f**) comparison of the thicknesses of pristine Ti foil (middle, thickness 127 μm), TiN-coated foil (left), and TiO_2_-coated foil (right).

Optical microscopy and SEM were used to examine the layer growth and surface modification of the Ti foil upon the thermal reaction. Figure 5 shows the optical microscope images of the fracture surfaces of the foil specimens taken at magnifications of 400~2000 times. Upon either the nitridation or the oxidation, clear boundaries were formed beneath both sides of the foil, and the outer layers became thicker with the increases in the heating temperature or the time. The TiO_2_ region was opaque and white, whereas the TiN and Ti_3_O regions appeared dense and metallic, consistent with the UV–Vis spectra. The conversions of titanium to oxide or nitride were accompanied by significant expansions along the thickness (Figure 5f), accordant with the unit cell volumes of Ti (35.3 Å^3^, *Z* = 2) [48], TiN (76.3 Å^3^, *Z* = 4) [51], and TiO_2_ (62.4 Å^3^, *Z* = 2) [49].

**Figure 6:**
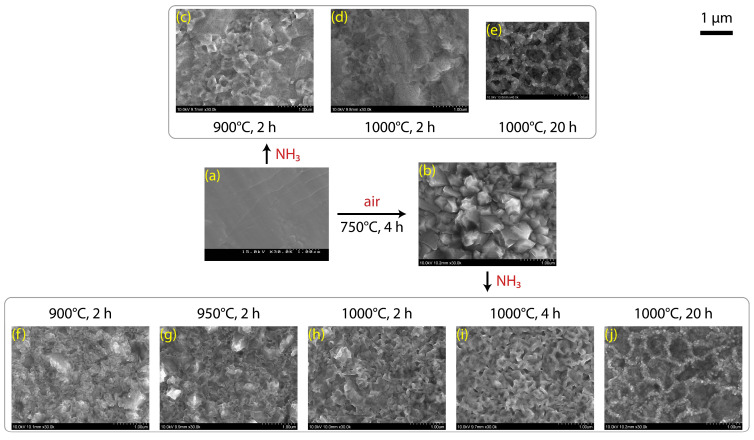
Plane-view SEM images of (**a**) Ti foil; (**b**) TiO_2_ coating; (**c**–**e**) TiN coatings prepared directly from the Ti foil, and (**f**–**j**) TiN coatings prepared from the TiO_2_ layer.

Figure 6 presents the SEM images showing the surface morphology of the TiO_2_ and TiN layers formed on the Ti foil. The pristine Ti foil had a smooth and flat surface (Figure 6a). Heating the Ti foil in air at 750 °C for 4 h resulted in a TiO_2_ layer formed of coarse grains with sizes of several hundred nanometers. Both the pristine Ti and the TiO_2_ layer were readily nitrided via ammonolytic heating, where the surface morphology evolves gradually with increases in temperature or time. The TiN layers formed by heating at 1000 °C for 20 h in NH_3_ had markedly wrinkled surfaces (Figure 6e,j), presumably because the nitride conversion expands the volume to disrupt the compact texture. Thus, the porosity of TiN layers can be controlled to some extent, which is relevant to various applicability such as in catalysts and electrodes.

### 3.4. Electrochemical Properties

Transition metal (Ti, V, Mo, etc.) nitrides and oxynitrides have attracted attention due to their potential electrochemical applications in energy storage [31,32]. In particular, the promising supercapacitor properties of titanium (oxy) nitride have been reported using various synthetic strategies. In many previous studies, TiN and TiO*_x_*N*_y_* were prepared in the powder form, focusing on a larger specific surface area via morphology control to increase the capacitance [36,39,41]. Those studies usually employed the slurry processing for preparing the electrodes. Alternatively, the active material was directly prepared on the conducting support by means of sputtering [38,43], hydrothermal treatment [55,56], electrochemical modification [34], and anodization [42] with the aim of improving the substrate contact. Specific capacitance (*C*_s_) is one of the primary figures of merit for assessing a supercapacitor. Table S1 shows a compilation of previously reported *C*_s_ values for various TiN-based supercapacitors where, it has to be noted, *C*_s_ depends significantly on the charge/discharge rate of GCD or the scan rate of CV.

**Figure 7:**
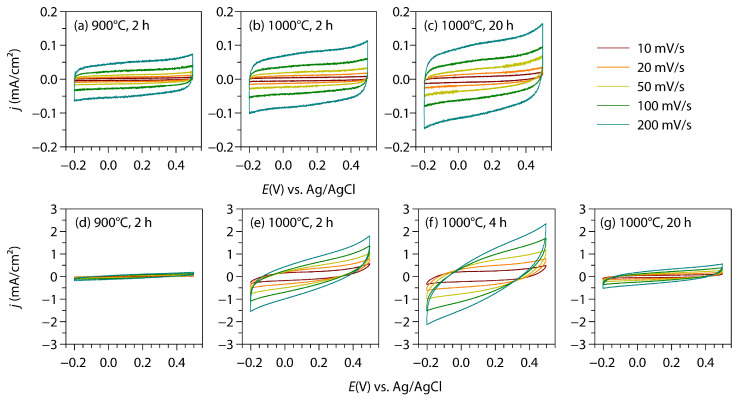
Cyclic voltammograms of (**a**–**c**) TiN coatings prepared directly from the Ti foil, and (**d**–**g**) TiN coatings prepared from the TiO_2_ layer. The ammonolysis temperature and time are shown in each panel.

**Figure 8:**
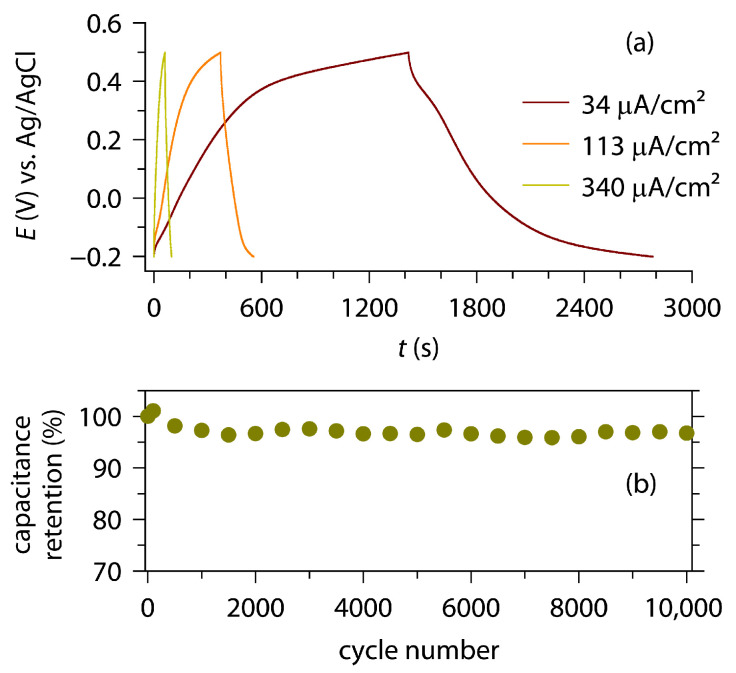
(**a**) Galvanostatic charge–discharge curves and (**b**) long-term stability test using cyclic voltammetry (200 mV/s) for the TiN coating prepared via ammonolysis of the TiO_2_ layer at 1000 °C for 4 h.

Figure 7 shows the CV diagrams, measured using two types of TiN coatings: prepared from the Ti foil (a–c) and from the TiO_2_ layer (d–g). The TiN coatings obtained from TiO_2_ display remarkably larger CV currents than those derived directly from Ti, implying the greater capacitance of the former. Among the TiN coatings obtained from TiO_2_, the highest electrochemical activity was observed from the sample prepared via ammonolysis at 1000 °C for 4 h. The capacitance behavior was examined also using GCD (up to 0.34 mA/cm^2^), which indicated the same trend as the CV investigation, that is, the greatest capacitance was observed from the TiN derived from ammonolysis at 1000 °C for 4 h, and whose capacitance behavior was further examined via a long-term durability test (CV, 200 mV/s). The GCD profiles are presented in Figure 8a, and the areal specific capacitance, *C*_s_, was calculated as given in Table 1. The *C*_s_ values observed in the present study (Table 1) are comparable to those of the previous studies (Table S1). For example, sputtered TiN films in different studies exhibited *C*_s_ values of 10.1 mF/cm^2^ (CV, 5 mV/s), 14 mF/cm^2^ (CV, 10 mV/s), and 27.3 mF/cm^2^ (GCD, 1 mA/cm^2^). Moreover, an extended CV test revealed that more than 95% of the capacitance was retained, even after 10,000 cycles of charge–discharge (Figure 8b). These observed electrochemical behaviors suggest that TiN layers can be thermally grown with widely different morphologies and capacitances, and they demonstrate the viability of metal foil-based supercapacitors. It is particularly notable that the present TiN supercapacitor exhibits comparable performances to those of sputtered specimens, presumably owing to the superior contact with the current collector.

## 4. Conclusions

The surface of the Ti foil was modified to various oxides and nitrides by adjusting heat treatment conditions such as temperature, time, and gas atmosphere. In particular, rutile TiO_2_ was formed by heating at >700 °C in air, while Ti_3_O was obtained by milder heating in a N_2_ flow or in static air. On the other hand, ammonolytic heating produced TiN and TiN*_x_* (*x* < 1), where the nitridation level depended mostly on the processing temperature. In all the above cases, the oxide or nitride coatings had clear borderlines with the inner Ti region, thus providing two-dimensional heterostructures of metal/semiconductor or metal/metal. Crystal structural evolutions upon the oxide and nitride conversions of Ti caused inevitable morphological changes that endowed varying degrees of porosity to the coated layer. The electrochemical CV and GCD tests revealed that the two-step treatments for the Ti-to-TiO_2_-to-TiN conversion provide superior capacitor properties. This study introduces a simple route to the two-dimensional heterostructures consisting of the electroactive material and conducting substrate, where the morphology of the former can be tailored by fine control of the experimental parameters. Also noted is that the limited reactivity of the dense metal can lead to metastable compositions such as Ti_3_O.

## Figures and Tables

**Table 1:** Areal specific capacitance of TiN derived from ammonolysis (1000 °C, 4 h) of TiO_2_ coating, based on CV and GCD.

CV		GCD	
v (mV/s)	*C*s (mF/cm^2^)	*j* (mA/cm^2^)	*C*s (mF/cm^2^)
10	37.0	0.034	66.2
50	19.6	0.045	57.7
100	13.0	0.113	29.8
200	9.0	0.340	17.6

## Data Availability

The data presented in this study are available on request from the corresponding author due to privacy.

## Supplementary Materials

The following supporting information can be downloaded at: https://www.mdpi.com/article/10.3390/ma18020380/s1, Table S1: Brief review of the specific capacitances (*C*_s_) per area (in F/cm^2^) or volume (in F/cm^3^) of various titanium nitrides; Figure S1: Powder XRD patterns of reagents, Ti foil, Ti powder, and TiO_2_ powder: Miller indices are based on the ICSD data: no. 52522 for Ti (*P*6_3_/*mmc*, *a* = 2.951 Å, *c* = 4.686 Å), and no. 16636 for TiO_2_ (*P*4_2_/*mnm*, *a* = 4.594 Å, *c* = 2.959 Å); Figure S2: Evolution of XRD patterns upon the heat treatment of Ti foil in N_2_, depending on temperature and time: Ti and Ti_3_O are both hexagonal and greater peak shifts are observed for (002) and (012) diffractions than (010) and (011) ones; Figure S3: XRD patterns corresponding to the conversions of (a) Ti foil to TiO_2_ to TiN and (b) TiO_2_ powder to TiN; Figure S4: Powder XRD patterns of Ti powder and heat-treated products in N_2_ or NH_3_, with different temperatures and times; Figure S5: UV–Vis diffuse–reflectance spectra of Ti and TiO_2_. Refs. [57,58,59,60,61,62,63] are cited in the supplementary materials.
