# Supplementary material for: Growth of Oxide and Nitride Layers on Titanium Foil and Their Electrochemical Properties
Source: Materials (Basel). 2025 Jan 15;18(2):380. doi: 10.3390/ma18020380 (PMC11766468; doi:10.3390/ma18020380)
Supplement: Supplementary file 1 [file materials-18-00380-s001.zip › materials-3427068-supplementary.pdf]

Supplementary Materials for

Growth of Oxide and Nitride Layers on Titanium Foil and Their Electrochemical Properties

Song Hyeon Kim and Young-Il Kim*

Department of Chemistry, Yeungnam University, Gyeongsan 38541, Republic of Korea

*Correspondence: yikim@ynu.ac.kr

Table S1. Brief review of the specific capacitances (C_s) per area (in F/cm^2) or volume (in F/cm^3) of various titanium nitrides.

Remark	C_s		Test condition	Ref.
TiN nanopillar on Ti, by anodization and hydrothermal treatment	120 F/cm^3		GCD 0.83 A/cm^3	S1
TiN nanowire	0.33 F/cm^3		GCD 2.5 mA/cm^3	S2
TiN on Si by sputtering		14 mF/cm^2	CV 10 mV/s	S3
TiN on Si by sputtering		27.3 mF/cm^2	GCD 1 mA/cm^2	S4
TiN on textured Si by sputtering		10.1 mF/cm^2	CV 5 mV/s	S5
TiN on CNT by sputtering		18.3 mF/cm^2	CV 1 V/s	S6
TiN on CNT by ALD		81 mF/cm^2	CV 100 mV/s	S7
TiN nanotube on Ti by anodization		3.14 mF/cm^2	CV 20 mV/s	S8
TiN nanoparticle by electrodeposition		69 mF/cm^2	CV 50 mV/s	S9
TiNbN bimetal by sputtering		59.3 mF/cm^2	GCD 1 mA/cm^2	S10
MoN_x on TiN nanotube		121.5 mF/cm^2	GCD 0.3 mA/cm^2	S11

S1. Qin, P. et al. Hierarchical TiN nanoparticles-assembled nanopillars for flexible supercapacitors with high volumetric capacitance. *Nanoscale* **2018**, *10*, 8728–8734.

S2. Lu, X. et al. Stabilized TiN nanowire arrays for high-performance and flexible supercapacitors. *Nano Lett.* **2012**, *12*, 5376–5381.

S3. Achour, A. et al. Titanium nitride films for micro-supercapacitors: Effect of surface chemistry and film morphology on the capacitance. *J. Power Sources* **2015**, *300*, 525–532.

S4. Wei, B. et al. Magnetron sputtered TiN thin films toward enhanced performance supercapacitor electrodes. *Mater. Renew. Sustain.* **2018**, *7*, 11.

S5. Sun, N. et al. TiN thin film electrodes on textured silicon substrates for supercapacitors. *J. Electrochem. Soc.* **2019**, *166*, H802-H809.

S6. Achour, A. et al. Hierarchical nanocomposite electrodes based on titanium nitride and carbon nanotubes for micro-supercapacitors. *Nano Energy* **2014**, *7*, 104–113.

S7. Kao, E. et. al., ALD titanium nitride coated carbon nanotube electrodes for electrochemical supercapacitors. 2015 Transducers-2015 18th International Conference on Solid-State Sensors, Actuators and Microsystems (TRANSDUCERS), IEEE; 2015, p. 498–501.

S8. Xie. Y. et al. Electrochemical capacitance performance of titanium nitride nanoarray. *Mater. Sci. Eng. B* **2013**, *178*, 1443– 1451.

S9. Ansari, S. et al. Electrochemical synthesis of titanium nitride nanoparticles onto titanium foil for electrochemical supercapacitors with ultrafast charge/discharge. *Sustain. Energy Fuels*, **2020**, *4*, 2480–2490.

S10. Wei, B. et al. All nitride asymmetric supercapacitors of niobium titanium nitride-vanadium nitride. *J. Power Sources* **2021**, *481*, 228842.

S11. Xie, Y.; Tian, F. Capacitive performance of molybdenum nitride/titanium nitride nanotube array for supercapacitor. *Mater. Sci. Eng. B* **2017**, *215*, 64–70.

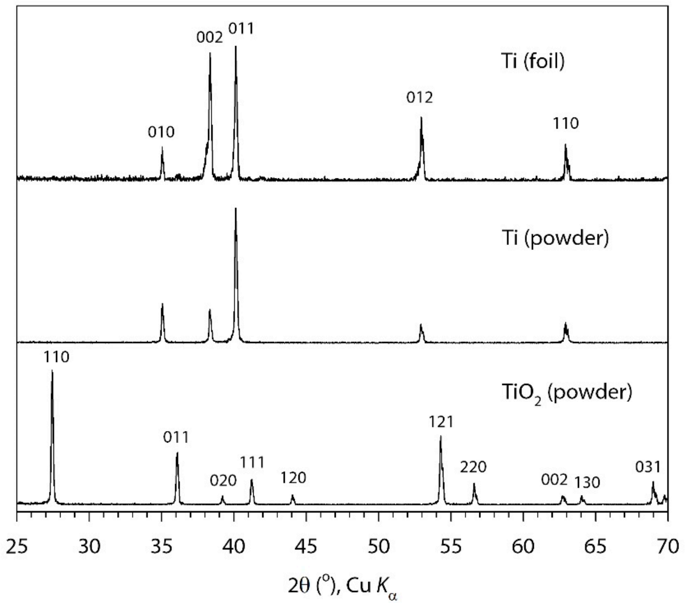

Figure S1. Powder XRD patterns of reagents, Ti foil, Ti powder, and TiO_2 powder: Miller indices are based on the ICSD data: no. 52522 for Ti ($P6_3/mmc$, a = 2.951 Å, c = 4.686 Å), and no. 16636 for TiO_2 ($P4_2/mnm$, a = 4.594 Å, c = 2.959 Å).

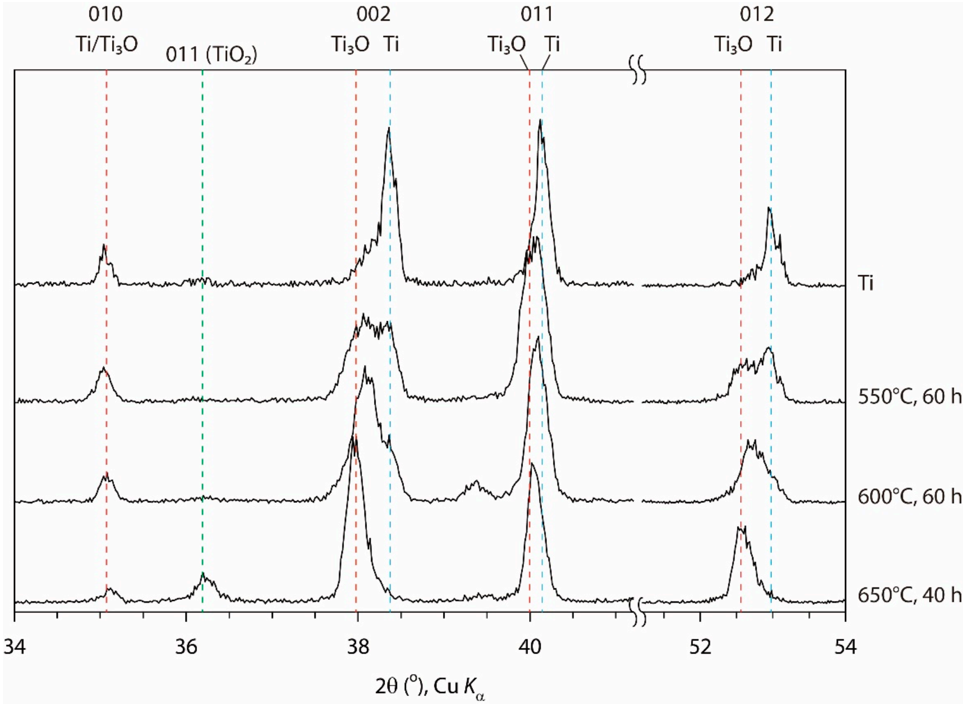

Figure S2. Evolution of XRD patterns upon the heat treatment of Ti foil in N_2, depending on temperature and time: Ti and Ti_3O are both hexagonal and greater peak shifts are observed for (002) and (012) diffractions than (010) and (011) ones.

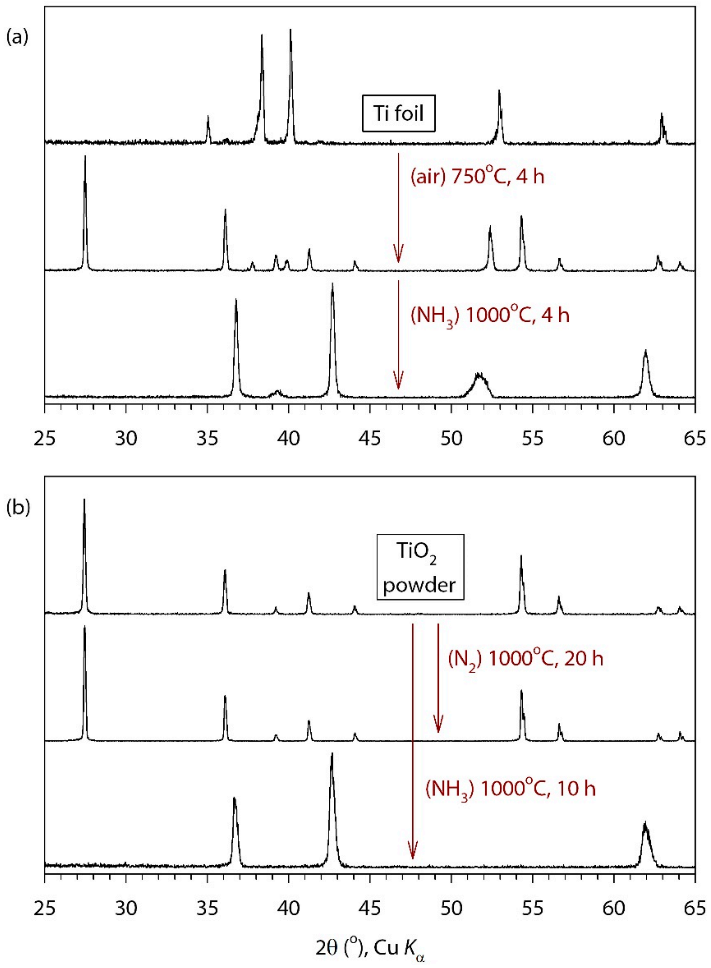

Figure S3. XRD patterns corresponding to the conversions of (a) Ti foil to TiO_2 to TiN, (b) TiO_2 powder to TiN.

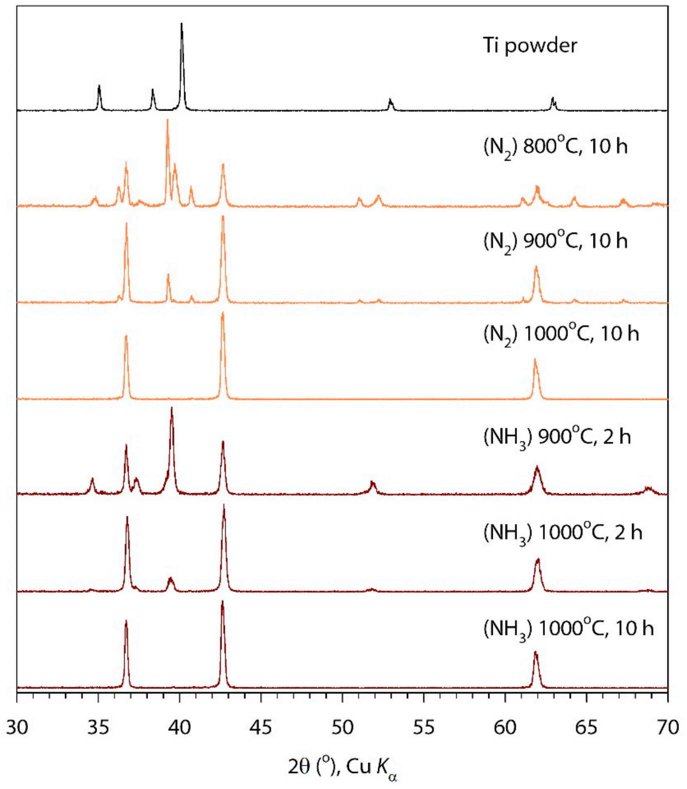

Figure S4. Powder XRD patterns of Ti powder and heat-treated products in N_2 or NH_3, with different temperatures and times.

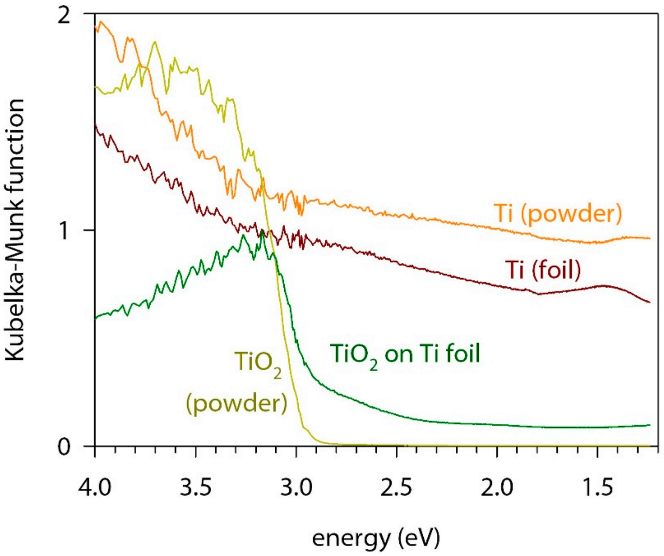

Figure S5. UV-Vis diffuse-reflectance spectra of Ti and TiO_2.
